# Semi-Targeted Metabolomics to Validate Biomarkers of Grape Downy Mildew Infection Under Field Conditions

**DOI:** 10.3390/plants9081008

**Published:** 2020-08-10

**Authors:** Kévin Billet, Magdalena Anna Malinowska, Thibaut Munsch, Marianne Unlubayir, Sophie Adler, Guillaume Delanoue, Arnaud Lanoue

**Affiliations:** 1 EA2106 “Biomolécules et Biotechnologies Végétales”, UFR des Sciences Pharmaceutiques “Philippe Maupas”, Université de Tours, 31 av. Monge, F37200 Tours, France; kevin.billet@univ-tours.fr (K.B.); magdalena.malinowska@univ-tours.fr (M.A.M.); thibaut.munsch@univ-tours.fr (T.M.); marianne.unlubayir@univ-tours.fr (M.U.); sophie.adler@orange.fr (S.A.); 2 Faculty of Chemical Engineering and Technology, Cracow University of Technology, 24 Warszawska St., 31-155 Cracow, Poland; 3 Institut Français de la Vigne et du Vin, 509 avenue Chanteloup, F37400 Amboise, France; Guillaume.DELANOUE@vignevin.com

**Keywords:** grape, downy mildew, semi-targeted metabolomics, infection biomarkers, polyphenols, stilbenoids, correlation network

## Abstract

Grape downy mildew is a devastating disease worldwide and new molecular phenotyping tools are required to detect metabolic changes associated to plant disease symptoms. In this purpose, we used UPLC-DAD-MS-based semi-targeted metabolomics to screen downy mildew symptomatic leaves that expressed oil spots (6 dpi, days post-infection) and necrotic lesions (15 dpi) under natural infections in the field. Leaf extract analyses enabled the identification of 47 metabolites belonging to the primary metabolism including 6 amino acids and 1 organic acid, as well as an important diversity of specialized metabolites including 9 flavonols, 11 flavan-3-ols, 3 phenolic acids, and stilbenoids with various degree of polymerization (DP) including 4 stilbenoids DP1, 8 stilbenoids DP2, and 4 stilbenoids DP3. Principal component analysis (PCA) was applied as unsupervised multivariate statistical analysis method to reveal metabolic variables that were affected by the infection status. Univariate and multivariate statistics revealed 33 and 27 metabolites as relevant infection biomarkers at 6 and 15 dpi, respectively. Correlation-based networks highlighted a general decrease of flavonoid-related metabolites, whereas stilbenoid DP1 and DP2 concentrations increased upon downy mildew infection. Stilbenoids DP3 were identified only in necrotic lesions representing late biomarkers of downy mildew infection.

## 1. Introduction

*Plasmopara viticola* (Berk. & M. A. Curtis) Bed. and De Toni is the causal agent of grape downy mildew and can severely affect the quality and quantity of vine production in temperate and wet climates [1]. Disease management strategies rely on multiple fungicide applications with potential harmful effects on human health and environment [2,3,4]. The ability to reduce pesticide use depends on multiple strategies including; resistance breeding, management practices, biocontrol, and precision viticulture [5,6,7].

Downy mildew oospores germinate during spring producing macrosporangia that release the asexual zoospores [8]. Early developmental stages including zoospore release, germ tube emission and entering through stomata, remain asymptomatic. At 4–5 days post infection (dpi) on the adaxial leaf surface appear the first visible symptoms called oil spots corresponding to green-yellow lesions [8]. At this stage, photosynthesis and resistance related genes are repressed in susceptible *Vitis. vinifera* L. cultivars [9] in association to a leaf source-to-sink transition [10]. Foliar lesions produce sporangia responsible for secondary infections. Later, oil spots extend as yellow circular spots and become necrotic when the sporangial production declines [8].

To date, biomarkers has been investigated in resistant cultivars through controlled greenhouse experiments with the aim to better understand the causes of incompatible interactions between *V. vinifera* and *P. viticola* [11]. In the resistant variety Bianca, downy mildew triggers foliar early changes (1–2 dpi) in primary metabolism including lipids, fatty acids, ceramides, amino acids, and sugars whereas from 4 dpi, it induces the plant defense metabolism namely phenylpropanoids, flavonols, and stilbenoids [12] including *E*-resveratrol, δ-viniferin, and ε-viniferin [13,14]. Early and high *E*-resveratrol biosynthesis has been observed in resistant cultivar [15]. Moreover, the amount of dimeric stilbenoids δ-viniferin and ε-viniferin in downy mildew infected leaves has been correlated to the resistance level [16]. Among the 60 stilbenoids described in *V. vinifera* L. [17,18], pterostilbene and δ-viniferin are the most active compounds against *P. viticola* [19] by inhibiting zoospore motility and sporulation [20]. Whereas most studies investigated resistance biomarkers for accelerated breeding purposes, the identification of biomarkers in susceptible varieties as new molecular phenotyping tool for viticulture precision remains poorly explored.

Metabolomics is a powerful technique that fill the gap between genomes and phenomes by predicting external phenotypes with the characterization of internal phenotypes based on metabolic variations under environmental changes [21]. To achieve this goal several metabolomics approaches have been developed. Whereas targeted metabolomics presents high quantitative reliability but covers only preselected metabolites, untargeted metabolomics presents the best coverage of metabolites but low reproducibility and substantial data processing. Semi-targeted metabolomics represents a mid-way strategy enabling a good compromise between metabolite coverage, reliability, effort in method construction and data treatments [22,23,24]. By the way, field-based studies using this strategy have been successfully applied to discriminate grape cultivars using extracts from berries or stems [25,26] and to finely describe the plasticity of grape metabolome in relation to terroir variations [27].

The aim of this study was to select biomarkers of natural grape downy mildew infection at the vineyard scale in leaves of a susceptible cultivar using UPLC-DAD-MS-based (Ultra Performance Liquid Chromatography coupled to Diode Array Detection and Mass Spectrometry) semi-targeted metabolomics. In particular, we evaluated metabolic changes in symptomatic leaves of *V. vinifera* L. cv. Malbec that expressed oil spots (6 dpi; days post infection) and necrotic lesions (15 dpi). Metabolomics profiling allowed the identification and the relative quantification of 47 metabolites that belong to both primary (amino and organic acids) and specialized metabolism including phenolic acids, flavonols, flavan-3-ols as well as stilbenoids with various degree of polymerization (DP). Unsupervised and supervised multivariate statistics were employed to reveal metabolic changes associated to the infection status. The identification of infection biomarkers was realized using results from both univariate (non-parametric one-way variance analysis) and multivariate statistics (VIP; Variable Importance in the Projection). Finally, correlation-based networks emphasized the structuration of grape metabolome when challenged by downy mildew. The present list of biomarkers might assist the developments of non-invasive phenotyping techniques to detect downy mildew at vineyard scale.

## 2. Results and Discussion

### 2.1. Metabolomics Profiling of Downy Mildew Infected Grape Leaves

Semi-targeted metabolomics method was developed specifically on grape leaf extracts; thus, extending previously established database dedicated to grape stem extracts [25]. Positive- (ESI^+^) and negative-ion (ESI^−^) electrospray ionization modes were used to identify molecular ions from downy mildew infected leaf extracts. As a result, a list of 47 analytes associated to retention time (RT), MS- and UV-spectra was established (Table 1). For 32 analytes, the putative molecular assignment has been confirmed by comparison with pure standards (level 1 in confidence of metabolite identification; [28]) including L-proline (m01), L-leucine (m02), L-isoleucine (m03), L-phenylalanine (m04), L-tyrosine (m05), L-tryptophan (m06), citric acid (m07), *E*-resveratrol (m08), *E*-piceatannol (m09), catechin (m10), epicatechin (m11), coutaric acid (m12), caftaric acid (m13), rhamnetin (m14), fertaric acid (m15), *E*-piceid (m17), kaempferol 3-*O*-glucoside (m19), pallidol (m20), *Z*-ε-viniferin (m23), *E*-ε-viniferin (m24), *E*-δ-viniferin (m26), kaempferol 3-*O*-glucuronide (m28), quercetin 3-*O*-glucoside (m29), quercetin 3-*O*-glucuronide (m30), myricetin 3-*O*-glucoside (m31), procyanidin B1 (m33), procyanidin B2 (m34), procyanidin B3 (m35), kaempferol 3-*O*-rutinoside (m38), quercetin 3-*O*-rutinoside (m39), *E*-miyabenol C (m41), and procyanidin C1 (m45). Other analytes were identified by comparison of elution order as well as MS and UV spectra obtained from literature describing grape metabolic composition (level 2 of identification). Peak m16 with an [M-H]^−^ ion at *m/z* 389 produced a daughter ion at *m/z* 227 (resveratrol) corresponding to a glucoside loss (−162 Da), and was therefore annotated as a resveratrol glucoside [29]. Peak m18 showed an [M-H]^−^ ion at *m/z* 441 and presented two fragments at *m/z* 289 (catechin unit) and *m/z* 169 (gallate unit) and was annotated as catechin gallate [30]. Peaks m21, m22, and m27 gave an [M-H]^−^ ion at *m/z* 453 with a fragment at *m/z* 265 [31]. The λ_max_ at 225 nm and 283 nm were characteristic of *Z*-stilbenoids isomers [14]; consequently, these 3 analytes were temporary assigned as *Z*-resveratrol dimers. Peak m25 (RT = 14.31 min) with a molecular ion [M-H]^−^ at *m/z* 453 was eluted between *E*-ε-viniferin (m24, RT = 13.24 min) and *E*-δ-viniferin (m26, RT = 14.88 min) and was assigned as *E*-ω-viniferin as previously reported [25,29]. The peak m32 showed a [M-H]- ion at *m/z* 479 with fragments at *m/z* 317 (-162 Da, loss of hexoside) and 271 and was annotated as myricetin hexoside [32]. Peaks m36 and m37 showed an [M-H]^−^ ion at *m/z* 577 and fragments at *m/z* 289, 425, and 407 characteristic for procyanidin B1 (m33), B2 (m34), and B3 (m35) and were provisionally assigned to procyanidins B4 and B5 [33]. Peaks m40, m42, and m43 presented an [M-H]^−^ ion at *m/z* 679 and daughter ions at *m/z* 585 and 491, they were temporally assigned as resveratrol trimers [29]. Peak m44 presented an [M-H]- ion at *m/z* 865 producing a daughter ion at *m/z* 576 (-289 Da, catechin unit loss) and was assigned as procyanidin trimer [34]. Peaks m46 and m47 produced [M-H]- ions at *m/z* 881 giving daughter ions at *m/z* 592 (-289 Da, catechin loss) and at *m/z* 305 (-576 Da, gallocatechin loss) and were annotated as prodelphinidin trimers [35].

After establishing the grape leaf metabolome, UPLC-MS analyses were achieved using selected ion monitoring (SIM) mode and the resulting SIM chromatograms were integrated. The corresponding relative quantifications of metabolites were used for further unsupervised and supervised multivariate statistics.

### 2.2. Unsupervised Multivariate Statistics for a Brief Overview of Metabolic Changes Upon Natural Downy Mildew Infection Under Field Condtions

**Figure 1:**
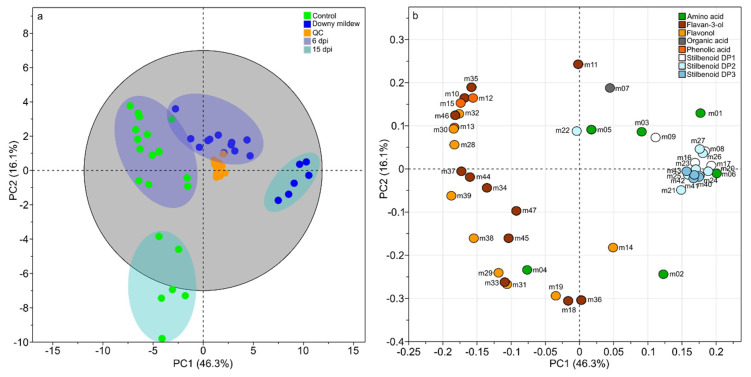
Unsupervised classification using principal component analysis (PCA) on the relative concentration of 47 metabolites from leaves of *Vitis vinifera* L. cv. Malbec upon downy mildew infection. Score plot (**a**). Dark blue ellipses: 6 days post infection (dpi). Light-blue ellipses: 15 dpi. Loading plot (**b**). Numbers indicate the ID of the compounds as given in Table 1.

A principal component analysis was performed as unsupervised analysis to show the differences in the metabolomics composition of grape leaves upon natural downy mildew infection at 6 and 15 dpi. The PCA score plot of the two first component explained 62.4% of the variation (Figure 1) with the first principal component (PC1) accounting for 46.3% and the second (PC2) for 16.1%. Projection on these two axes clearly separated samples into five groups (Figure 1a). Quality Control (QC) samples appeared well grouped and centered confirming the robustness of UPLC-MS measurements and low analytical variability (Figure 1a). The four other groups of samples were clearly discriminated according to infection status (control/downy mildew) and time following infection (6 and 15 dpi). Since control leaves at 6 and 15 dpi corresponded to the third and sixth positions from apex, respectively, the discrimination of these two groups of samples showed a change in the metabolomics composition according to leaf development. However the infection status was the main factor of variation with a projection of samples “downy mildew at 15 dpi” along PC1 (46.3%) and a projection of samples “control leaves at 15 dpi” along PC2 (16.1%). Whereas a slight overlap occurs between “downy mildew” and “control” conditions at 6 dpi, a clear discrimination was observed between the two groups at 15 dpi. The corresponding loading plot showed the variables responsible for such discriminations (Figure 1b) with an over-accumulation of stilbenoids DP1-3 in infected leaves (PC1 positive) and phenolic acids, flavan-3-ols, and flavonols in control leaves (PC1 negative). To a lesser extent, the abundance of some metabolites seemed associated to leaf development, for instance catechin gallate (m18), procyanidin B4 (m36), kaempferol 3-*O*-glucoside (m19), myricetin 3-*O*-glucoside (m31), and quercetin 3-*O*-glucoside (m29). To increase the separation of sample groups according to infection status and to identity infection biomarkers, we performed orthogonal partial least squares discriminant analysis (OPLS-DA) on the two data subsets (6 and 15 dpi) with the infection status (downy mildew or control) as discriminant variable.

### 2.3. Supervised Multivariate Statistics with “Infection Status” as Discriminant Variable

**Figure 2:**
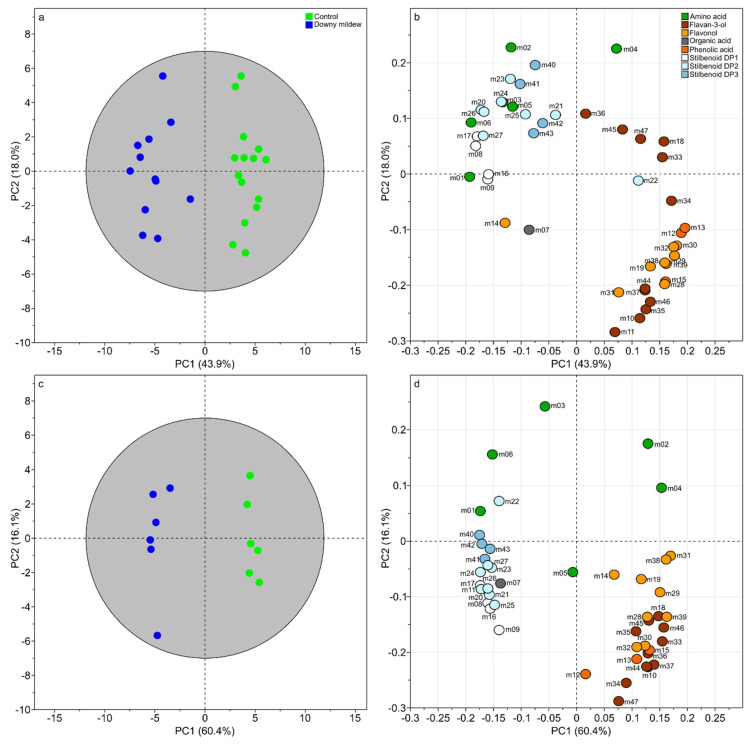
Supervised classification using orthogonal partial least squares discriminant analysis (OPLS-DA) with “infection status” as discriminant variable at 6 dpi (**a**,**b**) and 15 dpi (**c**,**d**). Score plot (**a**). Loading plot (**b**). Numbers indicate the ID of the compounds as given in Table 1.

The first model (diagnostic of the model: R^2^X_cum_ = 70.5%, R^2^Y_cum_ = 93% and Q^2^_cum_ = 86.3%) for the dataset corresponding to 6 dpi clearly separated samples according to the infection status with 67.3% of explained variance on the two first components (Figure 2a). The loading plot (Figure 2b) showed the variables responsible for the discrimination with an over-accumulation of stilbenoids DP1 (m08, m09, m16, and m17), DP2 (m20, m21, and m23–27), DP3 (m40–43), and several amino acids (m01–03, m05, and m06) infected leaves and an increase of phenolic acids (m12 and m13), flavan-3-ols (m10, m11, m18, m33–37, and m44–47), flavonols (m14, m19, m28–32, m38, and m39) and one amino acid (m04). The second model built with the dataset at 15 dpi showed even high degree of confidence (R^2^X_cum_ = 76.5%, R^2^Y_cum_ = 98.7% and Q^2^_cum_ = 97.3%) and an excellent separation of samples with PC1 and PC2 explaining 60.4% and 18% of variance, respectively (Figure 2c). The corresponding loading plot mainly confirmed previous observations on specialized metabolism regulations with an over-accumulation of stilbenoids DP1 (m08, m09, m16, and m17), DP2 (m20–27), DP3 (m40–43), and a decreased of phenolic acids (m12 and m13), flavan-3-ols (m10, m11, m18, m33–37, and m44–47), flavonols (m14, m19, m28–32, m38, and m39) in infected leaves. However, at 15 dpi, we noticed several changes in primary metabolism compared to 6 dpi. Some amino acids were induced in infected leaves (m01 and m06) when others were decreased (m02 and m04). Because many metabolites variations seemed to be associated to a specific metabolic pathway, we performed a correlation-based networks at 6 and 15 dpi.

### 2.4. Metabolic Network Analysis of Grape Leaves Upon Downy Mildew

**Figure 3:**
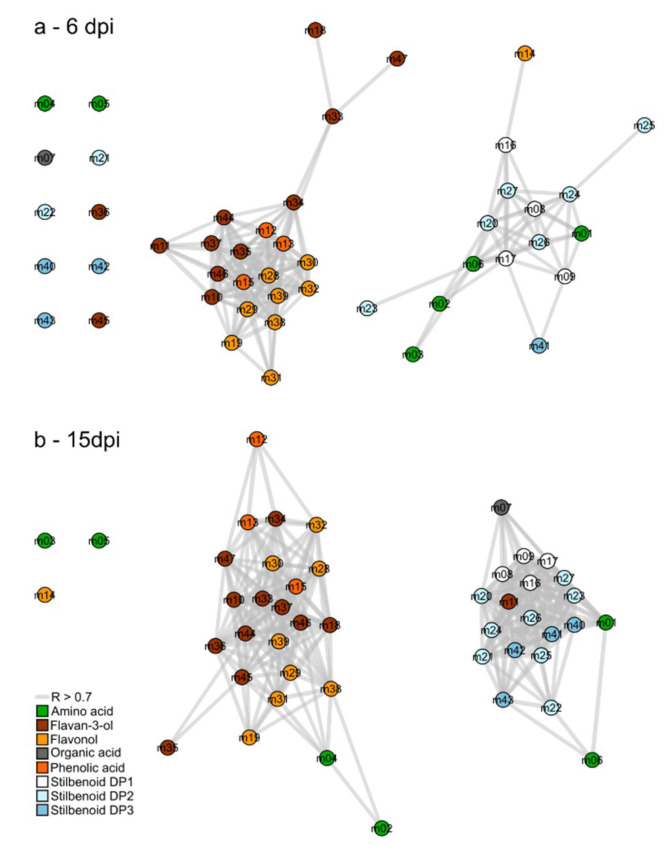
Correlation-based networks of metabolism from leaves of *Vitis vinifera* L. cv. Malbec upon downy mildew infection at 6 (**a**) and 15 dpi (**b**). Nodes numbers refers to the ID presented in Table 1 and edges to significant correlation of metabolite pairs (threshold: *R* > 0.7 and *p*-value < 0.05).

Correlation-based metabolic networks are relevant to reveal complex interactions between metabolites from complex high-throughput data. Spearman pair-wise correlations were calculated between the 47 metabolite amounts from control and infected leaves. Among the 1081 tested correlations, the two networks at 6 and 15 dpi depicted significant correlations (threshold: *R* > 0.7 and *p*-value < 0.05) by edges as multiple lines connecting the nodes (metabolites; Figure 3). As a result, the connectivity level was high on the network at 6 dpi with 164 significant positive correlations (Figure 3a) and even higher at 15 dpi (Figure 3b) with 292 significant positive correlations. 

Correlation-based networks showed that structurally related compounds were inter-correlated and clustered together. Moreover, both networks presented similar clustering topology that encompass two metabolite clusters. In the first cluster, metabolites mainly belonged to the flavonoid pathway including flavan-3-ols and flavonols, whereas in the second, metabolites were mainly associated to stilbenoid metabolism. A slight difference was observed between 6 and 15 dpi, concerning the position of amino acids. For instance, L-leucine (m02) showed a covariation with stilbenoids at 6 dpi and later with flavonoids (15 dpi).

### 2.5. Variable Selection for the Identification of the Infection Biomarkers

**Figure 4:**
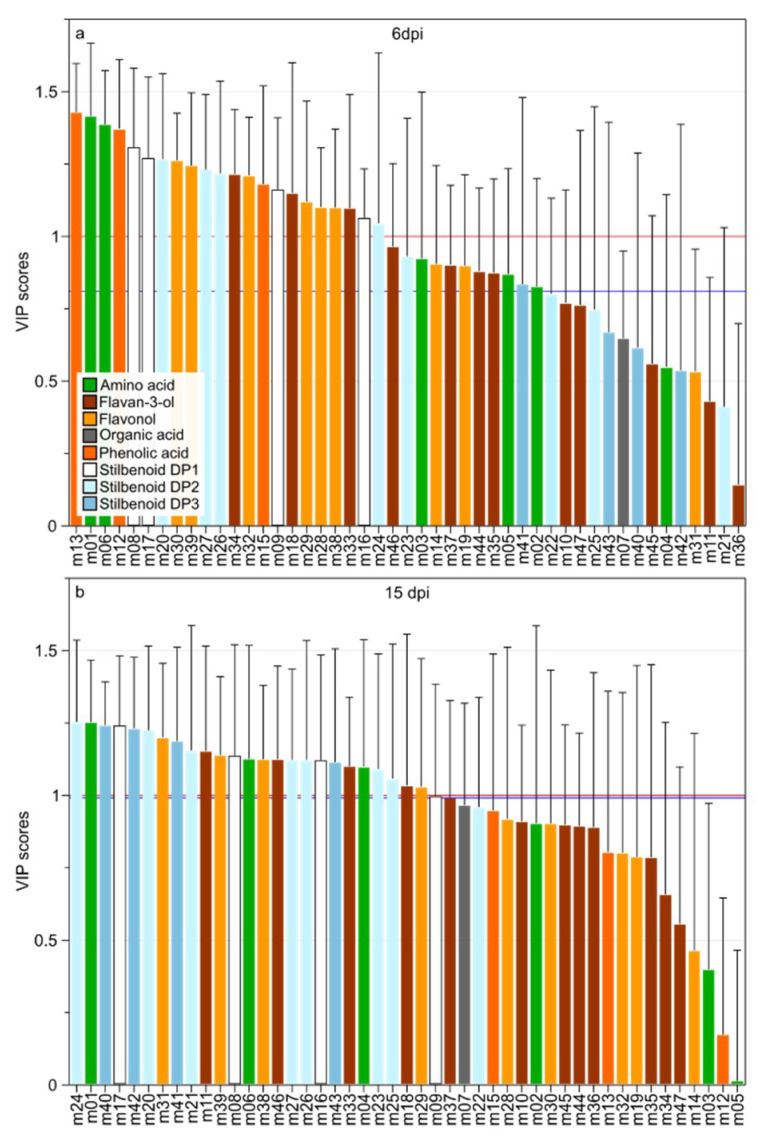
VIP-scores of the OPLS-DA models performed on oil spots symptomatic leaves data (6 dpi; **a**) and on necrotic symptomatic leaves (15 dpi; **b**) with a cutoff level of VIP > 1 (red lines) or depending on non-parametric statistical analysis (blue lines; 6 dpi = 0.81; 15 dpi = 0.99). Each variable was colored according to the polyphenol class and numbers indicated the ID of the compound as given in the Table 1.

Infection biomarkers were selected using both multivariate statistics (VIP; Variable Importance in the Projection) and univariate (non-parametric one-way variance analysis; Kruskal–Wallis test). Using the VIP method and a cutoff value of VIP > 1, 22 and 25 metabolite biomarkers were selected for 6 and 15 dpi, respectively (Figure 4). Kruskal–Wallis test allowed the selection of 33 and 27 metabolites for 6 and 15 dpi, respectively. The Figure 4 shows the ranking of metabolite variables according to VIP scores with the cutoff VIP > 1 (red line) and the limit of selected variables using Kruskal–Wallis test (blue line). The standard cutoff VIP > 1 is usually accepted with the goal to limit the number of false positive without impacting the true positive rate, however the choice of cutoff threshold depends on the data structure [36]. At 6 dpi, the Kruskal–Wallis test allowed the selection of 33 metabolites) corresponding to a cutoff value of VIP > 0.8 (Figure 4a). At 15 dpi, the two statistical methods selected variables corresponding to VIP > 1. 

At 6 dpi (oil spots), the 33 infection biomarkers belonged to amino acids (m01–03, m05, and m06), flavan-3-ols (m18, m33–m35, m37, m44, and m46), flavonols (m14, m19, m28–30, m32, m38, and m39), stilbenoids DP1 (m08, m09, m16, and m17), stilbenoids DP2 (m20, m23, m24, m26, and m27), stilbenoids DP3 (m41), and phenolic acids (m12, m13, and m15). At 15 dpi (necrotic lesions), the 25 selected metabolites belonged to amino acids (m01, m04, and m06), flavan-3-ols (m11, m18, m33, and m47), flavonols (m29, m31, m38, and m39), stilbenoids DP1 (m08, m09, m16, and m17), stilbenoids DP2 (m20–27) and stilbenoids DP3 (m40–43). The relative abundance of selected metabolites at 6 and 15 dpi was then evaluated.

### 2.6. Changes in Relative Concentration of the Selected Metabolites in Grape Leaves Upon Downy Mildew Infection

**Figure 5:**
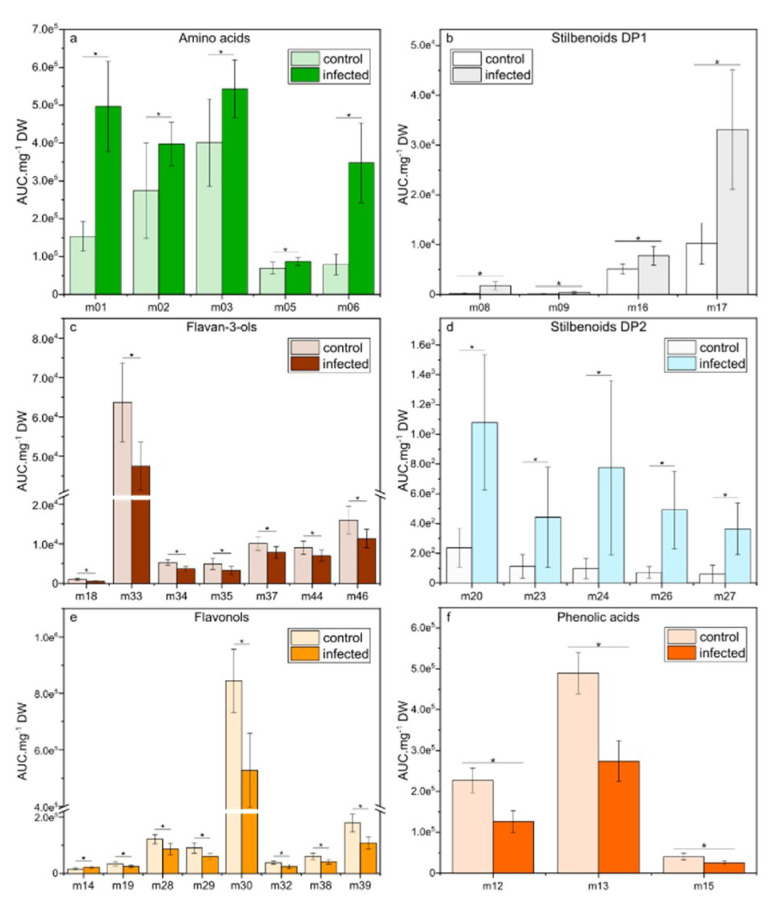
Relative concentrations of the selected metabolites in leaves of *Vitis vinifera* L. cv. Malbec upon downy mildew infection at 6 dpi. Data are mean values ± standard deviation (control: n = 15; infected: n = 12). Asterisk: significance difference from control (*p* < 0.05).

At 6 dpi, when oil spots appeared on the leaf surface, relevant metabolites (VIP-scores > 0.81) were either induced (amino acids and stilbenoids DP1-2) or repressed (flavan-3-ols, flavonols, and phenolic acids) compared to control conditions. Amino acids L-proline (m01), L-leucine (m02), L-isoleucine (m03), L-tyrosine (m05), and L-tryptophan (m06) presented significant (*p* < 0.05) higher content in infected leaves (Figure 5a), with a 3.2- and 4.4-fold increase for L-proline (m01) and L-tryptophan (m06), respectively. The stilbenoids DP1-2 *E*-resveratrol (m08), *E*-piceatannol (m09), resveratrol glucoside (m16), *E*-piceid (m17), pallidol (m20), *Z*-ε-viniferin (m23), *E*-ε-viniferin (m24), *E*-δ-viniferin (m26), and *Z*-resveratrol dimer3 (m27) were strongly induced in infected leaves (Figure 5b,d). As an example, pallidol (m20) and *E*-ε-viniferin (m24) presented a 4.5- and 7.9-fold increase in infected leaves, respectively. In the meantime, the flavan-3-ols catechin gallate (m18), procyanidin B1 (m33), procyanidin B2 (m34), procyanidin B3 (m35), procyanidin B5 (m37), procyanidin trimer1 (m44), and prodelphinidin trimer1 (m46) were decreased in infected leaves (*p* < 0.05, Figure 5c). A similar decrease was observed for the flavonols kaempferol 3-*O*-glucoside (m19), kaempferol 3-*O*-glucuronide (m28), quercetin 3-*O*-glucoside (m29), quercetin 3-*O*-glucuronide (m30), myricetin hexoside (m32), kaempferol 3-*O*-rutinoside (m38), and quercetin 3-*O*-rutinoside (m39; Figure 5e), except for rhamnetin (m14), which was slightly induced. Additionally, the levels of the phenolic acids; coutaric (m12), caftaric (m13), and fertaric acids (m15) were lower in infected leaves compared to control leaves (Figure 5f). 

**Figure 6:**
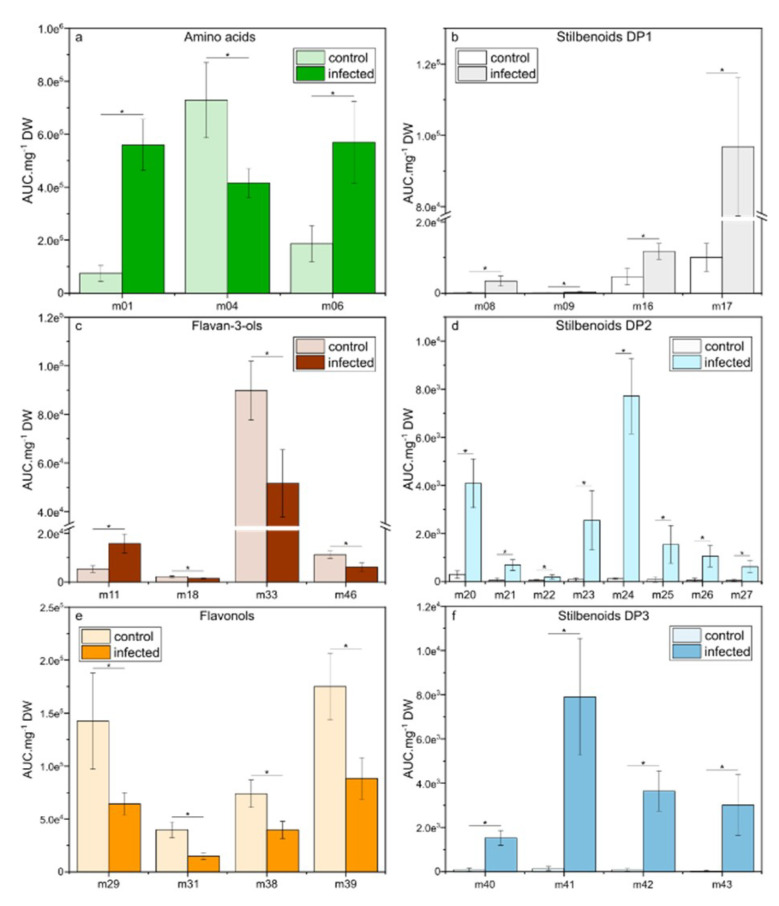
Relative concentrations of the selected metabolites in leaves of *Vitis vinifera* L. cv. Malbec upon downy mildew infection at 15 dpi. Data are mean values ± standard deviation (control: n = 6; infected: n = 6). Asterisk: significance difference from control (*p* < 0.05).

At 15 dpi, when necrotic spots appeared on the leaf surface, we observed a higher number of relevant metabolites (VIP-scores > 1) compared to 6 dpi and different metabolite trends were observed depending on metabolic pathways (Figure 6a–f). For amino acids, only L-proline (m01) and L-tryptophan (m06) remained induced, whereas a decreased of L-phenylalanine (m04) concentration was observed (Figure 6a). Within flavan-3-ols, only catechin gallate (m18), procyanidin B1 (m33), and prodelphinidin trimer1 (m46) remained decreased while epicatechin (m11) appeared newly induced. Concerning flavonols, only four metabolites were repressed (seven at 6 dpi), including quercetin 3-*O*-glucoside (m29), myricetin 3-*O*-glucoside (m31), kaempferol 3-*O*-rutinoside (m38), and quercetin 3-*O*-rutinoside (m39). Whereas phenolic acids were not anymore selected as relevant metabolites (VIP-scores > 1), the stilbenoid metabolism was strongly induced at 15 dpi with four stilbenoids DP1, eight stilbenoids DP2 and four stilbenoids DP3. The strongest increased was *E*-piceid (m17; 9.4-fold) for stilbenoids DP1, *E*-ε-viniferin (m24; 60-fold) for stilbenoids DP2, and *E*-miyabenol C (m41; 57-fold) for stilbenoids DP3.

## 3. Discussion

Metabolomics applied to vineyards aims to determine internal phenotypes of grapevine based on metabolite variations (metabotypes) depending on genotype, environment, and management practices. The identification of biomarkers from high-throughput metabolomic data represents a key step to convert large datasets of raw analytical data into relevant biological informations. While untargeted metabolomics provide a maximal metabolite coverage with a low reliability, targeted metabolomics provide an accurate quantification but on a limited number of known metabolites. UPLC-DAD-MS-based semi-targeted metabolomics represents an intermediate analytical approach that relies on a MS full-scan screening to identify unexpected metabolites offering in the meantime robust quantifications. In this study, we used semi-targeted metabolomics to screen downy mildew symptomatic leaves that expressed oil spots (6 dpi) or necrotic lesions (15 dpi) under natural infection in the field. A rapid MS screening of extracts from infected leaves enabled the identification of 47 metabolites including 6 amino acids, 1 organic acid, 9 flavonols, 11 flavan-3-ols, 3 phenolic acids, and stilbenoids with various degree of polymerization; 4 stilbenoids DP1, 8 stilbenoids DP2 and 4 stilbenoids DP3. Univariate (Kruskal–Wallis test) together with multivariate (VIP method) statistics allowed for the selection of 33 and 27 metabolites as relevant infection biomarkers at 6 and 15 dpi, respectively. Correlation-based networks clearly showed two metabolite clusters at 6 and 15 dpi corresponding to up and down variations following downy mildew infection.

In earlier studies, the metabolic profiling of downy mildew infected grape leaves was particularly focused on hybrid cultivars presenting partial resistance to *P. viticola* with the aim of determining resistance biomarkers for future grapevine breeding programs. Fifteen oligomeric resveratrol derivatives (stilbenoids DP2-4) were identified in infected leaves of an F1 hybrid of Merzling (a mid-resistant hybrid) and Teroldego a susceptible *V. vinifera* cultivar [11]. A study on stilbenoid accumulation in a segregating population for resistance to *P. viticola* showed that phytoalexin production (composition, level, and the delay of induction) was associated to the degree of resistance [37]. Recently, an extended metabolic profiling of infected leaves of the same genotype showed the production of 42 polyphenols including stilbenoids, flavonoids, and phenolic acids [38]. The combined use of LC-MS and GC-MS methods provided a higher metabolite coverage with 176 compounds from grape leaf extracts of the resistant cultivar Bianca including 53 metabolites modulated in response to *P. viticola* [12].

In the present study, the main purpose was the identification of infection biomarkers in susceptible *V. vinifera* cv. Malbec as new molecular phenotyping tool under field conditions. At 6 dpi (oil spots), we selected 33 infection biomarkers including 5 amino acids, 7 flavan-3-ols, 8 flavonols, 4 stilbenoids DP1, 5 stilbenoids DP2, 1 stilbenoid DP3, and 3 phenolic acids. At 15 dpi (necrotic lesions), 25 infection biomarkers were identified including 3 amino acids, 4 flavan-3-ols, 4 flavonols, 4 stilbenoids DP1, 8 stilbenoids DP2, and 4 stilbenoids DP3. In the susceptible cultivar Malbec the stilbenoid metabolism was induced upon downy infection, although stilbenoid biosynthesis and oligomerization did not confer resistance to *P. viticola*. We noticed the absence of tetrameric resveratrol derivatives (hopeaphenol, isohopeaphenol, *E*-vitisin B, and Viniferol E) in infected grape leaves while these compounds were found to be largely accumulated in grape canes of the same variety [25].

The metabolomics study showed that infection biomarkers were dependent of disease stage. While at 6 dpi, infection biomarkers mainly belonged to amino acids, flavonoids, and stilbenoids DP1-2, at 15 dpi the diversity and levels of stilbenoids DP2-3 were higher. We observed that stilbenoid monomers represented early phytoalexins (6 dpi) whereas oligomers were biosynthesized later (15 dpi). Especially, trimeric resveratrol derivatives represented biomarkers only at late infection stage when necrotic lesions were visible. It is consistent with previous observations where stilbenoid oligomerization occurred at advanced stages of downy mildew disease. In resistant cultivars, monomers (*E*-resveratrol, *E*-piceid) were early induced at 6–12 h after infection), dimers (*E*-ε-viniferin, and *E*-δ-viniferin) at 24–48 h when trimers and tetramers were accumulated at 96 h [12,13]. Previous studies reported higher anti-mildew activities of stilbenoids DP3 compared to DP1-2 [20,39]. Interestingly, the susceptible cultivar Malbec was also able to induce resveratrol trimers even though their concentrations rose too late and too weak to confer resistance.

Correlation-based networks (Figure 3) combined to histograms of VIP (Figure 5 and Figure 6) emphasized several metabolic changes under downy mildew infection. Within primary metabolism, all amino acids were induced at 6 and 15 dpi, except L-phenylalanine that was unchanged at 6 dpi and became repressed at 15 dpi. A general increase of primary metabolism has been reported in *P. viticola* infected leaf tissues associated to the induction of several amino acids [9,38,40]. The present increase of amino acid biosynthesis supports previous description of a source-to-sink transition in infected leaves of susceptible cultivars [10]. The decrease of the L-phenylalanine pool as precursor of the general phenylpropanoid pathways corresponded to the induction of stilbenoid metabolism. Phenylpropanoid metabolism showed finely tuned metabolic regulations with an over-accumulation of stilbenoid metabolism while flavonoid metabolism was repressed. The two closely related polyketide synthases; chalcone synthase (CHS) and stilbene synthase (STS) competed for the same substrates; *p*-coumaroyl-CoA and 3 units of malonyl-CoA. In infected leaves the metabolic flux balance was then preferentially routed toward stilbenoid metabolism. Interestingly, such antagonistic crosstalk was observed in resistant cultivars at early stages of infection (16–72 h post infection) and not in susceptible varieties [41]. Metabolomics data at 6 and 15 dpi suggested that in susceptible cultivars, the shutdown of flavonoid branch pathway occurs too late to result in substantial induction of effective defense molecules.

At the vineyard scale, high-throughput phenotyping techniques like semi-targeted metabolomics analyses provide biomarkers or groups of biomarkers to assist the development of non-invasive phenotyping techniques. Disease detection still remains challenging for automated field phenotyping and the most advanced techniques rely on multispectral sensors combined to machine learning [7,42]. Amino acids, flavan-3-ols, and stilbenoids were the main groups of biomarkers identified in the present study and such metabolic targets might orientate the choice of adapted sensors for plant phenotyping. Using conventional laboratory spectrophotometer, the blue-violet auto-fluorescence of stilbenoids was locally monitored in downy mildew infected grape leaves [43,44]. Near-infrared (NIR) spectroscopy was assessed as non-invasive method to estimate amino acid contents in grape berries [45]. Integrative methodology combining metabolomics and plant phenotyping using UAV equipped with optical sensors has been proposed to assess water stress in vineyards [46]. Recently, grapevine disease detection using a deep learning approach from Unmanned Aerial Vehicle (UAV) images offers promising perspectives for decision support systems [47]. 

## 4. Materials and Methods 

### 4.1. Plant Material and Growth Conditions

**Figure 7:**
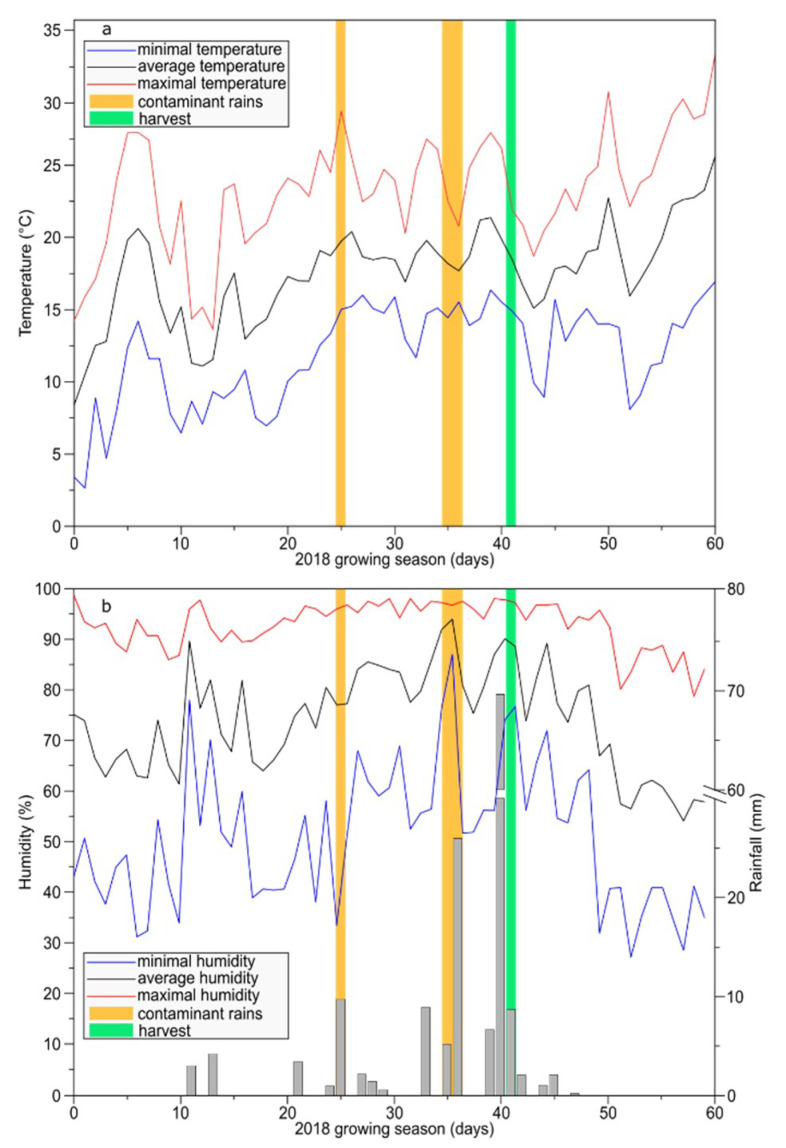
Vineyard climatic conditions during May and June 2018. (**a**) Temperature. (**b**) Humidity. Yellow bars: days of contaminant rains (26 May and 5–6 June 2018). Green bar: day of leaf harvest (11 June 2018).

The study was performed during vintage 2018 on 40-year-old grapevines from the school for Viticulture of Amboise (France; 47◦39′59.46′′N, 0◦97′61.54′′E). Vines of *Vitis vinifera* L. cv Malbec were planted on a calcareous soil at a spacing of 1 m (within row) × 1 m (between rows) corresponding to 6,666 vines ha^−1^. The vines were conducted with a double Guyot pruning system under the climate of the Loire Valley, a temperate oceanic climate. Two adjacent plots were subjected to organic management with similar practices except for copper-based fungicide treatments. On the first plot, copper treatments corresponding to 1 kg ha^−^^1^ were applied (control leaves), whereas no copper applications was applied to the second plot allowing natural downy mildew infections (infected leaves). The collection of leaves was realized on 11 June 2018, following two contaminant rains that took place 6 and 15 days before (Figure 7). Infected leaves corresponding to 6 dpi (oils spots, 12 replicates) were located on grape shoots at the 3rd position (from the apex) of fully expanded leaf [48]. Infected leaves corresponding to 15 dpi (necrotic lesions, 6 replicates) were older and located at 6th position on grape shoots. The corresponding control leaves were harvest at 3rd and 6th position on grape shoots from the adjacent plot without copper treatment. Days of contaminant rains were determined using climatic condition data associated to “Potential System^®^” method. As a result, oil spot symptoms observed on leaves on 3rd position from apex resulted from contaminant rains on 5/6 June 2018 (rainfall: 5 and 26 mm; average humidity: 92% and 94%; average temperature: 18.2 and 17.7 °C, respectively), consequently we determined that leaves with oil-spot lesions corresponded to 6 dpi. Necrotic lesions observed on the leaves on 6th position from apex resulted from contaminant rains on 26 May 2018 (rainfall: 10 mm; average humidity: 77%; average temperature: 19.8 °C), then the leaves with necrotic lesions corresponded to 15 dpi.

**Figure 8:**
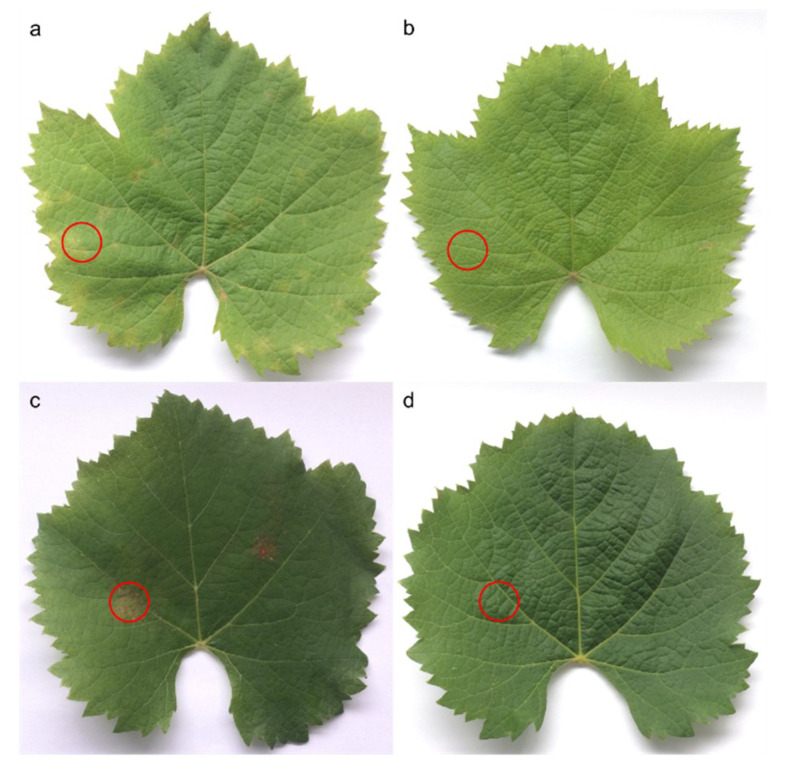
Leaves of *Vitis vinifera* L. cv. Malbec from 3rd (**a**,**b**) and 6th (**c**,**d**) positions from the apex on grape shoots. Downy mildew infected at 6 (**a**) and 15 dpi (**c**). Control (**b**,**d**). Circles indicate dissected zones.

Necrotic areas were dissected and stored in liquid nitrogen, whereas equivalent areas were harvested on control leaves (Figure 8). Following lyophilization, dried leaves were ground in a ball mill (Retsch MM200 mixer mill, Retsch GmbH, Haan, Germany) during 4 × 30 s at 30 Hz. Twenty milligrams of dried powder were extracted in a 1 mL solution of methanol–water (80:20; *v/v*) assisted by sonication (AL04-04, Advantage-Lab GmbH, Darmstadt, Germany) for 30 min. Finally, samples were centrifuged at 18,000 g for 10 min and stored at −20 °C until chromatographic analyses.

### 4.2. Chemicals

Acetonitrile and methanol were purchased from Thermo Fisher Scientific (France). A Millipore Milli-Q system (Merck Millipore, Molsheim, France) provided ultrapure water. L-leucine, L-isoleucine, L-phenylalanine, L-tyrosine, L-tryptophan, L-proline, citric acid, *E*-resveratrol, *E*-piceatannol, catechin, epicatechin, coutaric acid, fertaric acid, *E*-piceid, kaempferol 3-*O*-glucoside, and kaempferol 3-*O*-glucuronide were obtained from Sigma-Aldrich (St. Louis, MO, USA). Caftaric acid was purchased from Carbosynth (Compton, Berkshire, UK). Rhamnetin, *E*-ε-viniferin, kaempferol 3-*O*-rutinoside, quercetin 3-*O*-glucoside, quercetin 3-*O*-glucuronide, myricetin 3-*O*-glucoside, procyanidin B1, procyanidin B2, procyanidin B3, procyanidin C1, and kaempferol 3-*O*-rutinoside were purchased from Extrasynthèse (Genay, France). Pallidol and *E*-δ-viniferin were graciously provided by Dr. Mary-Lorène Goddard (EA-3991, Université de Haute-Alsace, Colmar, France). *E*-miyabenol C was obtained from Ambinter (Paris, France).

### 4.3. UPLC-MS Analyses

The UPLC-MS system consisted in an ACQUITY™ Ultra Performance Liquid Chromatography system coupled to a photo diode array detector (PDA) and a Xevo TQD mass spectrometer (Waters, Milford, MA) equipped with an electrospray ionization (ESI) source controlled by Masslynx 4.1 software (Waters, Milford, MA, USA). A Waters Acquity HSS T3 C18 column (150 × 2.1 mm, 1.8 μm) ensured the analytes separation with a flow rate of 0.4 mL min^−1^ at 55 °C. The injection volume was 5 μL. The mobile phase was made of 0.1% formic acid in water (solvent A) and 0.1% formic acid in acetonitrile (solvent B). The chromatographic separation was performed using an 18-min linear gradient from 5 to 50% solvent B followed washing and column reconditioning in 8 min. MS detection was performed in both positive and negative modes. The capillary voltage was 3000 V and sample cone voltages were 30 and 60 V. The cone and desolvation gas flow rates were 60 and 800 Lh^−1^, respectively. Metabolomics profiling was performed according to retention times, MS and UV spectra and comparison with standards or data from literature (Table 1). For relative quantification, UPLC-MS analyses were performed in selected ion monitoring (SIM) mode and the resulting SIM chromatograms were integrated using the ApexTrack algorithm with a mass window of 0.1 Da and relative retention time window of 0.2 min followed by Savitzky–Golay smoothing (iteration = 1, width = 1) using Targetlynx software. The resulting peak integrations and retention times were finally visually examined. The robustness of measurements and analytical variability were evaluated through a series of quality control (QC) samples prepared by pooling all samples and injected before, during, and after the batch. Samples were randomly injected independently from leaf position of infection status.

### 4.4. Statistical Analysis

Raw data of relative concentrations are available in Table S1. Multivariate analysis (MVA) was performed using SIMCA P+ version 12.0 (Umetrics AB, Umeå, Sweden). Variables were mean-centered and unit-variance scaled prior to MVA. Principal Component Analyses (PCA) was used as unsupervised MVA, to visualize differences and similarities among samples as well as to confirm robustness and analytical variability (QCs projection). Orthogonal Partial Least Square-Discriminant Analysis (OPLS-DA) was used as supervised method to maximize the difference between infected and control leaves. The quality of the OPLS-DA models was estimated with R^2^*X* (cum) the cumulative modeled variation in the X matrix (metabolomics variables), R^2^*Y* (cum) the cumulative modeled variation in the Y matrix (infected and control status), and *Q*^2^ (cum) the predictive ability.

Univariate statistical analysis was performed using R Statiscal Computing software (v3.6.2) [49]. As homoscedasticity (Bartlett test) and normality (Shapiro–Wilk test) assumptions were not reached, we performed non-parametric tests. We assessed multiple comparison of metabolite concentrations between infected and control leaves using Kruskall–Wallis test (pgirmess package with R). For correlation-based network, pair-wise Spearman correlation coefficients within 47 metabolites were calculated for all the 1081 metabolite pairs in R using the “cor” function and the significance of the correlation were obtained with the “cor.test” function. Significant correlation (R > 0.7) were visualized using “igraph” package (v1.2.4.2) [50] and the “graph.adjency” function. Layout of the network was adapted with the Fruchterman–Reingold algorithm [51].

## 5. Conclusions

A semi-targeted metabolomics approach was used to perform the field-metabotyping of downy mildew infected leaves that exhibit oil spots (6 dpi) or necrotic lesions (15 dpi) symptoms. As a result, 47 metabolites were identified belonging to both primary (amino and organic acids) and specialized metabolism (phenolic acids, flavan-3-ols, flavonols, and stilbenoids DP1-3). The selected infection biomarkers were specific to disease symptoms with 33 and 27 relevant metabolites at 6 and 15 dpi, respectively. In a nutshell, the amino acid metabolism was induced, whereas differential metabolic regulations were observed for polyphenols with a strong induction of stilbenoid metabolism associated to a down regulation of flavonoid metabolism. In a context of agriculture precision, high-throughput phenotyping techniques such as field-metabolomics might provide biomarkers or groups of biomarkers to assist the development of non-invasive phenotyping techniques such as multispectral or hyperspectral imaging.

## Figures and Tables

**Table 1:** List of polyphenols identified in grape leaves of Malbec cultivar.

Peak	Retention Time (min)	Phenolic Class	Compound Assignment	Molecular Ion	Product Ions	λmax (nm)	Authentication
m01	1.07	Amino acid	L-proline	116 [M+H]^+^	70		Standard
m02	1.67	Amino acid	L-leucine	132 [M+H]^+^	86		Standard
m03	1.80	Amino acid	L-isoleucine	132 [M+H]^+^	86		Standard
m04	2.78	Amino acid	L-phenylalanine	166 [M+H]^+^	120		Standard
m05	1.46	Amino acid	L-tyrosine	182 [M+H]^+^	165		Standard
m06	4.12	Amino acid	L-tryptophan	205 [M+H]^+^	188		Standard
m07	1.44	Organic acid	Citric acid	191 [M-H]^−^	111	327	Standard
m08	10.48	Stilbenoid DP1	*E*-resveratrol	227 [M-H]^−^	143, 185	305, 317	Standard
m09	8.72	Stilbenoid DP1	*E*-piceatannol	243 [M-H]^−^	159, 201	322	Standard
m10	5.05	Flavan-3-ol	Catechin	289 [M-H]^−^	203, 123	229, 278	Standard
m11	6.20	Flavan-3-ol	Epicatechin	289 [M-H]^−^	203, 123	229, 278	Standard
m12	4.97	Phenolic acid	Coutaric acid	295 [M-H]^−^	163, 149		Standard
m13	3.92	Phenolic acid	Caftaric acid	311 [M-H]^−^	179, 149		Standard
m14	3.15	Flavonol	Rhamnetin	315 [M-H]^−^	165, 121		Standard
m15	5.40	Phenolic acid	Fertaric acid	325 [M-H]^−^	193, 149		Standard
m16	6.55	Stilbenoid DP1	Resveratrol glucoside	389 [M-H]^−^	227, 185	279	Moss et al. (2013) [29]
m17	7.96	Stilbenoid DP1	*E*-piceid	389 [M-H]^−^	227, 185	279	Standard
m18	7.86	Flavan-3-ol	Catechin gallate	441 [M-H]^−^	289, 169		Vrhovsek et al. (2012) [30]
m19	9.34	Flavonol	Kaempferol 3-*O*-glucoside	449 [M-H]^−^	287, 153		Standard
m20	9.72	Stilbenoid DP2	Pallidol	453 [M-H]^−^	265, 359	232sh, 285	Standard
m21	10.54	Stilbenoid DP2	*Z*-resveratrol dimer1	453 [M-H]^−^	428, 265	225, 283	Püssa et al. (2006) [31]
m22	11.41	Stilbenoid DP2	*Z*-resveratrol dimer2	453 [M-H]^−^	428, 265	225, 283	Püssa et al. (2006) [31]
m23	12.87	Stilbenoid DP2	*Z*-ε-viniferin	453 [M-H]^−^	347, 359, 225	204, 285	Standard
m24	13.24	Stilbenoid DP2	*E*-ε-viniferin	453 [M-H]^−^	347, 359, 225	225sh, 323	Standard
m25	14.31	Stilbenoid DP2	*E*-ω-viniferin	453 [M-H]^−^	347, 359, 225	225sh, 325	[25,29]
m26	14.88	Stilbenoid DP2	*E*-δ-viniferin	453 [M-H]^−^	347, 359, 225	225sh, 327	Standard
m27	15.80	Stilbenoid DP2	*Z*-resveratrol dimer3	453 [M-H]^−^	428, 265	225, 283	Püssa et al. (2006) [31]
m28	9.26	Flavonol	Kaempferol 3-*O*-glucuronide	461 [M-H]^−^	205	265, 347	Standard
m29	8.40	Flavonol	Quercetin 3-*O*-glucoside	463 [M-H]^−^	301	256, 354	Standard
m30	8.27	Flavonol	Quercetin 3-*O*-glucuronide	477 [M-H]^−^	301	254, 355	Standard
m31	7.28	Flavonol	Myricetin 3-*O*-glucoside	479 [M-H]^−^	317, 271	262, 300sh, 353	Standard
m32	8.27	Flavonol	Myricetin hexoside	479 [M-H]^−^	317, 271	262, 300sh, 353	Figueiredo-González et al. (2012) [32]
m33	4.53	Flavan-3-ol	Procyanidin B1	577 [M-H]^−^	289, 425, 407	280, 313	Standard
m34	5.63	Flavan-3-ol	Procyanidin B2	577 [M-H]^−^	289, 425, 407	279	Standard
m35	4.83	Flavan-3-ol	Procyanidin B3	577 [M-H]^−^	289, 425, 407	279	Standard
m36	5.33	Flavan-3-ol	Procyanidin B4	577 [M-H]^−^	289, 425, 407	279	Ehrhardt et al. (2014) [33]
m37	6.86	Flavan-3-ol	Procyanidin B5	577 [M-H]^−^	289, 425, 407	279	Ehrhardt et al. (2014) [33]
m38	9.03	Flavonol	Kaempferol 3-*O*-rutinoside	593 [M-H]^−^	285, 255	265, 348	Standard
m39	8.11	Flavonol	Quercetin 3-*O*-rutinoside	609 [M-H]^−^	447, 301	256, 354	Standard
m40	13.36	Stilbenoid DP3	Resveratrol trimer1	679 [M-H]^−^	585, 491		Moss et al. (2013) [29]
m41	13.57	Stilbenoid DP3	*E*-miyabenol C	679 [M-H]^−^	573, 451		Standard
m42	13.98	Stilbenoid DP3	Resveratrol trimer2	679 [M-H]^−^	585, 491		Moss et al. (2013) [29]
m43	14.78	Stilbenoid DP3	Resveratrol trimer3	679 [M-H]^−^	585, 491		Moss et al. (2013) [29]
m44	5.13	Flavan-3-ol	Procyanidin trimer	865 [M-H]^−^	664, 576, 289		Monagas et al. (2006) [34]
m45	6.37	Flavan-3-ol	Procyanidin C1	865 [M-H]^−^	664, 576		Standard
m46	4.31	Flavan-3-ol	Prodelphinidin trimer1	881 [M-H]^−^	592, 305		Teixera et al., (2016) [35]
m47	5.95	Flavan-3-ol	Prodelphinidin trimer2	881 [M-H]^−^	592, 576		Teixera et al., (2016) [35]

## Supplementary Materials

The following are available online at https://www.mdpi.com/2223-7747/9/8/1008/s1. Table S1: Raw data of relative concentrations.
