# Supplementary material for: Semi-Targeted Metabolomics to Validate Biomarkers of Grape Downy Mildew Infection Under Field Conditions
Source: Plants (Basel). 2020 Aug 10;9(8):1008. doi: 10.3390/plants9081008 (PMC7465342; doi:10.3390/plants9081008)
Supplement: Supplementary file 1 [file plants-09-01008-s001.pdf]

infection status	dpi	m01	m02	m03	m04	m05
downy mildew	6	67369.5707			44583.7342	9198.88182
downy mildew	6		65148.8961			
downy mildew	6	66832.343	50842.5615	68701.2568	66635.9632	10671.3021
downy mildew	6	55005.1269	47467.6427	58623.3249	54148.3376	10917.8768
downy mildew	6		68572.8646	80732.529		10771.909
downy mildew	6	52009.0979	49241.569	90321.54	65198.8725	11715.6484
downy mildew	6	60974.7698	61890.8224	77120.4737	68959.1119	
downy mildew	6				44915.6986	
downy mildew	6				44931.1924	
downy mildew	6	67478.1629	51089.7348	66349.3056	53414.1414	8826.34626
downy mildew	6	54464.5164				9197.91377
downy mildew	6		62061.4884	82016.7849	59343.9593	11875.7798
downy mildew	15	123480.679	87350.0612	66526.1005	60371.7324	8899.87946
downy mildew	15	93142.3641	78230.6658	74175.3668		11902.5102
downy mildew	15				45762.0731	
downy mildew	15	106731.328				11021.9782
downy mildew	15		68016.0714	63448.0645	52724.6832	9709.45134
downy mildew	15	72901.9413	60801.5503	51808.7325	46685.9777	
control	6		14711.1913	25914.2211		3762.10264
control	6	9109.64744	14428.2852	21259.0687	31257.5503	4298.80854
control	6	11783.7853	13479.4818	21506.8609	36689.0232	4164.96
control	6	13022.2028		27304.7059	45817.8554	5062.92471
control	6	11983.0398				
control	6			21258.0979	40767.9937	4577.93233
control	6	12847.7336			49004.7094	4916.6047
control	6	11134.4291				3067.83859
control	6	13706.7426	12026.9911	28549.3613	32097.3156	3865.44011
control	6	9988.66216	19023.3784	23009.7246		
control	6	9664.02198	14988.2518	25412.6239	35516.6185	4952.57557
control	6	13984.4898	13940.1658	26011.8399	30078.2143	3467.1899
control	6		17763.3078	24249.2734	38110.2094	
control	6	10524.9117				
control	6	13272.5561	17721.5954	33399.8101	48300.259	6851.26773
control	15	5084.67838	57678.9213		79923.1599	5534.18782
control	15	5254.87341		17002.2888		6178.37527
control	15		42457.1755	24595.1998	50495.4338	
control	15		51330.3598	32993.2897	64934.5976	4898.31327
control	15	6687.66391				
control	15	7051.3234	47439.9594	27830.3031	59830.4063	4367.9586

m06	m07	m08	m09	m10	m11	m12
	6771.08354		37.3769697	13058.3452	1495.70475	17733.8479
58549.8423	6972.94757	209.711121	69.231215	12484.0976	1529.72009	12997.865
55850.4918		237.483934	25.673224	11416.1578	1336.69541	13000.3843
38020.2983	4752.56223	217.105685	39.7417259	9945.13645	1011.67645	13946.0169
		298.380556	41.8836111	11555.8094	1051.48167	15694.9595
37213.1637	6554.1068	163.047113	44.8310309	14276.0245		13647.202
52981.4178	7199.76726		64.6702105	12151.6012	1144.63242	11698.0732
	4827.11624		16.6596471	12431.3447	1486.00435	13191.4633
	5657.55152		19.38	11168.2978	1567.49548	12606.6892
34216.9066	5408.17	123.57798	36.4817172	11517.4677	1334.27626	16173.1818
33344.6388	4740.79578	226.605528	78.1553769	11388.1101	1458.33106	13597.299
	6884.19553		93.7446512	12172.2427	1359.7386	15730.4767
		612.713913	63.4269565	8668.1862	2114.51598	11292.7726
75792.303	7000.88696	559.374783	61.9363043			
40220.4084	6436.96782	617.627327	66.0020792	8986.46584	1947.87129	
	8330.34612		35.2457416	8331.18124	2339.95627	9999.96565
62347.9782	7312.64332	530.743226	24.5147465	7702.55253	2053.06544	11713.6464
85801.5084	7119.79441		42.6131844		1772.50235	11962.2791
3961.55695	3049.16579	4.43857868	6.08142132	11169.8953	1371.50244	16744.9715
5839.60462	2832.52905	8.0198995	5.41909548		642.014774	12284.2753
	3639.16693	2.27055276	4.35487437	10765.5826		17649.4472
4774.13186	3631.22471	8.63823529	3.43156863	10981.7203	1108.42127	14423.5891
	4345.54172	4.37606061	6.41747475	7381.66192	785.162424	20851.5152
3415.45233		18.9259361	16.6601826	8452.37233	794.943014	14061.875
	3971.4676	4.0551	14.1436	10038.107	1149.2372	17161.9141
2781.94131	3065.09206	21.1356784	10.4908543	9087.85879	1182.9995	15797.1985
	3733.90492	19.6217486	2.43169399		1313.26645	18376.6564
4481.15503		8.04875676	9.02551351	8221.77622	680.417405	17220.1909
4359.64774		10.5593396	16.0488679	8673.78906	878.143396	17007.2479
3771.42143	4069.60224	22.8090816	4.19	9473.32194	1039.3799	15854.7577
3962.5008	3784.7434	1.0483	1.8063	9147.3148	872.8264	14660.9203
	4095.57972	27.3759813	0.97663551	8766.02953	829.482617	19237.0108
9981.51249		10.8798895	0.37955801			14494.0677
20754.9286	2827.55117	3.63817259	1.86314721	6985.89624	557.060102	
		9.17923077	8.04978022	5535.8122	447.074505	
11803.6245		9.0052459	8.00983607	8114.92831		5313.50536
20302.4588	2755.54537	29.2091707	16.778439	7553.36888	591.70722	8535.99844
	2684.49598	0.51652174	6.38902174	8004.61283		7894.56772
14613.5063	2421.4271	10.4384	4.5918	7534.8773	517.3362	8259.1828

m13	m14	m15	m16	m17	m18	m19
30608.1629	3347.14444	3296.48121	1064.08293	3090.41172	75.5162626	2939.98556
32081.2793	3085.49654	2878.97505	921.030654	4047.4872	76.6464486	2769.71178
29616.2295	2193.78415	2252.9118	870.239454	4602.07049	51.7620765	2327.89617
29741.8528		2702.04985	771.345076	4540.51239	80.0739086	3124.26234
33206.9994	2950.90491	2901.6862	1035.05083	5306.64278	73.1788889	
33152.339	2763.68773	2969.46299	771.49567	2807.31454	52.0680412	3127.1466
32163.9375	2302.76684	2932.79358	1155.27979		93.1048421	
33012.0147		2425.40718			49.6474118	2212.15694
26510.9735	2554.97088	2588.74636	852.514747	2580.84009	58.0940092	3046.72304
31104.6622	2725.40323	3051.64596	796.027879	2450.85404	62.5244444	2811.93303
28891.2029	2940.44693	2911.14754	864.365628	3862.68412	52.7659296	2365.89548
30084.2936	2839.31209	3216.19981			76.2364651	2515.63972
30374.358		2313.0063		16093.9335	177.904239	6372.26098
35285.839	3429.09011	2580.56152			191.112391	5270.61315
34690.9592		2146.74604	1523.6802	13641.1958	179.050198	6314.57842
30421.3278	3569.04938	2773.13589	1576.48268	12426.9819		
25696.9585	3006.71871	2396.05023	1630.63991	12997.1241	238.94341	5264.51576
25543.1459	2787.8086	2028.82246	1240.13676	10825.3693	172.591732	6729.77564
33170.3553	846.550457	3127.37076	302.247411	432.432589	70.9106599	2309.04883
30024.799	846.03397	2092.30231	262.071658	490.646734	49.0045226	2285.64543
34604.3342	791.995678	3141.46	247.756181	297.024925	68.5903518	
35139.1391		2976.54206	466.186765		77.0812745	2494.90941
42052.8378		2531.79091			104.291717	2647.45263
29015.5622	1211.53361	2363.28128	436.681461	898.050502	84.8523288	2523.37132
36721.425	786.953	3125.2535	303.9163	380.5218	72.2403	2588.4029
33253.2004	1073.5407	2563.80945	283.472462	598.582412	25.9456281	2364.41166
38730.8504			432.111694	717.462842	51.369071	2749.24754
37453.7636	1322.68108	2901.31568	466.662162	757.15373	107.060108	1908.25232
31003.2547	1008.8484	2760.55792	308.666321	729.069245	63.1074528	2802.80462
35158.0677	832.422653	3243.82551	256.117449	700.041122	102.912449	2906.09776
35305.7531	910.7755	2550.7598	341.3525	452.7268	80.5295	2055.5383
41898.2506		2408.00636			117.536729	1852.90944
37086.6782	1069.56088	3470.03691	375.087182	665.4	80.3116022	2784.15812
30903.7563	2044.04173	1942.54741	286.48203	552.981929	193.63066	5147.83431
		1990.36593	315.08	726.385275	163.932857	
21758.5075	2224.07978	1823.56645			205.768743	10362.769
28500.7225	3294.0118	2054.89063	413.407317		193.788976	5816.19132
23067.6376	2035.51717	2082.65174	308.322391	649.252717	208.169348	10972.3438
26321.8344	3193.4891	1815.8801	362.7094	869.004	180.1968	6017.9281

m20	m21	m22	m23	m24	m25	m26
83.2193939	10.0154545	10.3754545	13.3046465		3.9759596	25.8667677
129.82215	4.66738318	8.84906542	52.6142056	130.902243	21.874486	59.0605607
200.667869	6.7	2.05169399	73.7040437	96.3742077	36.7935519	111.947213
153.367208	11.2826396	13.9760406	87.4749239	119.409645	43.737868	51.6585787
229.797685		6.84861111	171.515			
90.7684536	9.61814433	6.62010309	31.3853608	57.95	10.2369072	62.3115464
199.908632	6.27094737	0.64315789	65.5412632	68.8314737	11.7266316	80.8581053
37.8643529	6.73294118	19.7456471	5.63035294	30.0004706	9.66058824	13.9871765
52.4181567	3.19870968	7.59041475	18.6419355	29.7282028	9.72036866	11.0592627
62.7018182	1.22161616	10.5353535	28.8036364	26.9108081	20.3236364	32.4466667
99.9844221	5.26050251	1.70462312	19.3080402	64.0488442	47.6772864	46.480804
209.470047	0.40837209	8.86902326	89.7892093		46.1406512	101.785209
590.612391		35.6291304	514.532826		144.122174	93.5008696
642.234457	118.574891	28.4794565	570.07413	1271.8637	261.842717	
594.876337	107.150891			1053.14406	249.48198	136.01901
491.234928	118.110239	21.2107177	432.827177	1368.45952	225.937895	135.030718
639.193733		42.4369585	420.98682	1202.39152		
551.178101	120.22324	34.5591061		932.581453		102.601341
6.74111675	1.66020305	7.85583756	5.72233503	10.3121827	8.72345178	4.15451777
11.9846231	7.71326633	20.5162814	0.56572864	16.3620101	12.279196	7.2920603
0.70180905	4.4319598	2.40030151	20.7474372	4.08100503	5.59567839	2.40361809
39.0943137	2.03519608	17.0134314	9.39254902	2.96892157	0.43215686	4.07029412
35.0145455	7.60707071	13.0252525	16.2777778	9.94171717	15.0809091	11.2476768
19.2179909	2.14703196	12.4170776	3.4796347	2.76310502	2.69579909	1.8113242
10.3185	1.4317	20.4887	11.8783	8.4329	6.1592	2.7852
7.23035176	3.21065327	24.3226131	4.02683417	7.60201005	10.9092462	6.38884422
15.1805464	0.77879781	3.35081967	3.87693989	7.25453552	13.7521311	4.84371585
17.9428108	0.62227027	20.1269189	2.78183784	9.41091892	1.93362162	4.27167568
21.3365094	0.85245283	4.94443396	8.44575472	9.64103774	9.53688679	0.12783019
19.3804082	6.9727551	2.87867347	1.50244898	4.40244898	6.04122449	4.69867347
14.6574	1.2513	16.6615	14.3105	1.969	1.661	8.1462
35.3290654	1.41261682	17.6838318	18.3514953	5.82018692	17.1414019	10.9378505
10.9994475	5.03756906	18.1137017	6.65325967	0.95767956	0.37370166	6.40762431
13.2261929	5.64720812	10.0417259	4.23989848	17.2532995	4.10030457	8.48619289
24.9434066	0.09483516	5.8721978	6.8367033	10.1643956	3.6232967	14.2530769
39.9215301		3.22262295	15.0970492	10.6219672	21.515847	1.59781421
34.6174634	2.43512195	2.41326829	6.5395122	14.289561	0.17209756	6.44907317
15.256413	3.64369565	10.1028261	6.56217391	10.0128261	0.25108696	3.14032609
24.6318	2.7081	6.6298	8.2803	6.0088	17.3348	2.4765

m27	m28	m29	m30	m31	m32	m33
27.3007071	11150.8381	7006.12687	61067.4242		2754.36616	5738.89677
40.4114019	10495.0519	6772.51897	61115.9463	2101.50467	2691.57178	6535.27234
83.9713661	9368.60568	6418.74022	62647.5683	1629.01246	2960.06022	6712.74033
34.5033503	8772.66731	7782.59117	55182.1891	1985.64761	2473.6935	5517.51269
90.3446296	9369.19852	7332.83417	58042.2106	1873.84454	2785.13278	6465.37185
41.723299	10946.7228	7919.94928	61448.8789	1969.44845	2841.97794	6364.86186
55.1802105	10134.7047		63991.8224		2941.78947	5328.04811
	9841.05882	6276.40259	75472.9559	1434.20059	3749.58047	
35.1169585	11727.2055	6669.59677	55929.3722	2189.68894	2561.533	4541.11032
18.1430303	10297.7778	6052.54576	57519.9369	2173.87192	2827.56768	4230.14364
29.96	8968.6809	5682.66251	55347.4372	2060.05126	2377.0792	5591.4793
59.4187907	8970.532	7581.2093	58541.6221		2391.5814	6220.84521
100.518913	8436.36891	9267.19511	56571.8376	1559.46761	2538.55489	7108.69228
		8975.90696	67477.8193		2733.1562	8264.6
64.949703	8787.61297	8860.41921	56624.8762	2001.25327	2529.83832	8590.69228
83.3450718			57328.4989		2247.58019	7425.26766
89.4052535	10674.7675	7694.55714	53963.8192	1748.47659	2368.69438	6649.18488
48.2241341	9837.15084	9497.36905	56792.514	2095.58726	2526.06994	6113.27911
0.68375635	8354.16406	7257.16132	63274.8034	1646.79675	3029.57523	4130.97046
11.741608	7281.30653	6143.66442	55430.6093	1342.03186	2452.89578	3672.94523
0.16160804	9541.99829	7895.26151	69845.1571	1549.91638	3052.48683	4020.74864
3.91421569	8759.32676	7239.21029	65917.9106	1750.02873	3492.60382	5178.16137
12.8035354	10874.5943	6852.62939	66777.0266	1676.41121	3206.5801	
2.62374429	8685.13772	6258.40822	54514.9601	1587.25954	2453.72201	4990.68347
1.7862	8683.243	6312.075	63077.6125	1410.2535	2784.9664	3888.0137
2.46432161	8198.19799	6172.69588	46833.3448	1249.86472	2175.78121	
10.6903825	11030.52	7395.76328	72490.041	1687.06842	3284.31738	3872.87016
2.18281081	9048.26854	5844.14778	69810.5811	1352.32086	3103.91827	5645.69557
2.15735849	8512.27443	7465.88368	52529.1923	1928.56368	2448.52443	5090.49047
8.68418367	9103.21592	6978.06602	60890.236	1732.34061	2991.61949	4411.20255
0.8634	8959.8641	5877.9641	64744.8688	1402.73	2707.501	5610.4535
0.83028037	8744.02673	5315.01495	63254.2173	1456.93682	2820.88131	
3.45723757	9885.89006	6722.4642	72382.8108	1832.45536	3154.51359	4820.62884
7.98558376	8985.5401	9466.35949	58727.0685			8297.30964
7.00692308	8633.99978	8555.10648	59300.1305	2710.91176	2249.09022	6213.63407
6.60885246	6548.47596		46479.1326	3158.32503	1798.52077	8112.55552
7.76419512	9504.68449	11437.6319	50021.7135	3707.44585	1767.10439	8068.35141
1.17978261	9345.27087		48540.1597		1947.00891	8373.05283
0.7388	10206.5977	12023.9766	50665.5844	3691.2441	2293.001	8060.7414

m34	m35	m36	m37	m38	m39	m40
441.415152	392.584545	70.800404	1008.46596	4870.79545	13358.0493	2.72808081
510.075514	372.642243	30.8547664	1042.6486	4693.48131	12791.9758	3.5335514
	354.226667	45.6381421	897.70459	4047.44153	10887.7161	10.547541
388.481523	308.159898		744.711675	5245.01147	13770.4204	22.9232487
498.170185	293.14463	38.0500926	977.223519	5088.94824	11757.3307	
462.727113	413.22	43.2101031	1185.82247	5434.46361	14650.4848	14.9916495
402.313579	364.461895	35.7767368	810.164211		14890.75	20.1056842
380.085765	396.290706			3948.99447	11702.9385	6.63670588
361.32129	452.091797		965.227281	5327.7106	13316.1651	2.42599078
	302.880909		826.272828	4358.34596	11871.5688	13.1775758
400.392864		39.500804	958.939196	4126.78744	10217.9908	12.1061307
460.334233	393.034326	42.9334884	1014.88605	4379.30633	11437.7289	33.0296744
472.916739		63.9295652				240.116087
	223.300109	70.8568478	1083.85098	6485.76217	14898.5887	308.173152
655.569604			1023.53792	4900.49891	12829.3008	
604.476268	155.327464	83.3860287	943.206124		15813.9458	232.330718
539.628111	188.303502	47.5666359	855.97023	5864.3988	11468.2251	239.351152
479.167598	126.710726	63.3065922		5300.48659	12331.7685	223.252291
323.279695	426.976853		681.185076	4042.67208	14658.7374	2.18690355
		30.0176884	502.259397	3943.16623	12253.2585	4.63326633
410.46	459.11005		710.528643	5465.08955	15399.876	1.39427136
395.701373	347.483627	18.4901961	738.609118	4767.69912	14138.6857	6.64186275
		56.7560606	817.069293	5216.27525	15099.8927	4.81565657
358.585753	378.943288	48.1821005	688.414886	4207.2474	11266.753	1.66374429
366.6037	394.3516	11.0199	691.9783	3763.8145	12496.3117	6.6543
322.556181	433.313467		788.150553	4652.93065	11020.8872	1.12271357
412.458907	457.841202	10.2284153	965.730055	4903.21683	15195.6062	5.31912568
442.180973	297.606811	27.0715676	694.275459	3946.73189	11435.8928	6.41535135
343.060189		50.1384906	725.791132	5036.45604	14931.0658	
423.355306	420.220408	19.9394898	713.374184	4369.49776	13847.3421	2.1
487.0797	354.4927	13.6009	805.9552	4169.0902	11583.4422	1.546
	305.768692	42.4382243	854.879346	4319.8671	11962.8242	
430.004199	416.116022	12.4581215		4639.82044	13602.7866	7.27922652
	209.438883	62.2352284	877.045482	5348.56558	12738.3359	3.2986802
422.29044	132.467802	71.8520879	704.515714		13108.9698	14.7195604
352.926557	216.926885	93.2073224	853.971585	5427.62339	16457.8518	8.25617486
477.974634	167.186732	85.0798049	894.024585	7388.96498	15956.9344	9.56380488
401.089674			866.980761		17946.1498	5.19619565
473.4168		86.8833	865.0687	7545.3406	15823.4531	2.0335

m41	m42	m43	m44	m45	m46	m47
12.2512121	7.56424242		820.084242	150.171313	1424.08859	292.6
55.078972	18.2230841	34.9975701	1002.62458	166.738879	1485.51019	225.76972
83.4863388	50.4762842	50.4414208	963.291913	213.236721	1160.15454	372.96153
123.38467	55.1439594		712.10802	168.019898		338.012893
			797.735926		1258.78019	344.683704
47.6065979			1024.33289	163.111959		384.29299
19.3787368		26.0355789		111.640421	1170.02853	318.365684
	9.50447059	18.8702353			1158.94612	
	15.4069124	29.3588018	749.034747	131.001106	1345.54682	
33.6533333	13.7885859			112.431717	1228.23192	219.895556
44.5141709	20.558593	32.7515578	972.059799	210.362714	1348.13276	323.563618
147.606326		53.2671628	924.258698	188.558791	1612.7213	359.276837
		236.139348	702.110109		694.663696	257.524022
1210.1513	618.951196	391.5525		169.78337		
		293.325545	984.590792	186.888218	925.764653	
1186.11072	598.350431	434.686316	896.863062	197.390431	1006.62823	336.846603
1288.59954	538.178986		864.943226	133.194194	787.381198	297.007373
	663.816872				647.138436	264.209162
3.58700508	8.30812183	8.93126904	678.214924	96.7879188	1280.53462	252.335431
6.89467337	10.7327638		441.918392	94.9937688		178.31608
7.60482412		8.74090452	725.745025	155.774774	1319.07799	175.669548
6.64186275			582.111863	152.381275	1284.8048	281.376667
8.5569697			714.764141	164.76303	1035.81879	379.766566
15.4247489	9.1683105	11.5726941	655.686667	130.26137		289.41242
3.3688			672.3899		1237.9205	171.8136
14.8226131	10.9328643	8.95175879	728.719095		1216.69126	
4.03803279			837.52776		1444.39497	185.835628
11.3405405	11.8598919		637.088757	117.682378		161.703135
10.0203774		9.10528302	666.441981	126.916509	1097.20962	386.85434
1.52428571	16.8639796		650.003367	108.403878	1434.99153	262.918878
9.3039		6.2391	642.1625	141.0215	1142.2023	370.9216
11.1176636			718.611402		1257.40607	
1.33834254			744.92895	138.964088	1545.86641	291.841326
3.2986802	8.10690355	8.16446701	827.040203	171.977157	1036.44873	318.016244
6.74384615	2.72		559.549011		874.250549	262.860989
11.8708197	12.2210929		795.36623		902.047869	265.18623
24.5730732	3.85492683		788.391707	200.603512	985.246829	361.03961
6.95434783	3.0301087	7.52956522	794.039022	196.005435	1031.06457	304.451957
18.3746	13.0491		861.9894	139.5611	1062.6646	302.3116
